# Depressed visual field and mood are associated with sleep disorder in glaucoma patients

**DOI:** 10.1038/srep25699

**Published:** 2016-05-11

**Authors:** Masahiko Ayaki, Daisuke Shiba, Kazuno Negishi, Kazuo Tsubota

**Affiliations:** 1 Department of Ophthalmology, Keio University School of Medicine, Tokyo, Japan; 2 Shinseikai Toyama Hospital Eye center, Imizu, Japan

## Abstract

The aim of the present study was to evaluate sleep and mood disorders and related ocular parameters in glaucoma patients. We focused on visual fields and the retinal nerve fibre layer, because decreased circadian photoreception by damaged intrinsically photosensitive retinal ganglion cells is suspected in glaucoma. A cross-sectional study was performed on 140 subjects: 69 with glaucoma and 71 normal controls. Individuals with cataract, dry eye, or retinal pathology were excluded from the study. Participants completed the Pittsburgh Sleep Quality Index (PSQI) and Hospital Anxiety and Depression Scale (HADS) and underwent comprehensive ophthalmological examinations for glaucoma. Patients with advanced glaucoma had significantly worse PSQI scores than normal controls (*P* < 0.05). Stepwise multivariate linear regression analysis revealed PSQI was significantly correlated with the mean deviation in the worse eye, the number and frequency of medications, and anxiety and depression subscores of the HADS after adjustment for age and sex (*P* < 0.05). We did not find a significant correlation between PSQI scores and the thickness of retinal nerve fibre layer. In conclusion, the subjective sleep quality of glaucoma patients was correlated with visual field loss and mood status.

The primary pathology of glaucoma is damage to retinal ganglion cells (RGCs), including intrinsically photosensitive RGCs (ipRGCs), which are involved in circadian photoreception to regulate homeostasis in the entire body^1^,^2^. Photoreception by the ipRGCs modulates a non-visual response to light associated with sleep, circadian rhythm, headache, photophobia, and alertness. Irradiance of ipRGCs with short-wavelength light depresses melatonin secretion in a dose-dependent manner, and this pathway is a major determinant of sleep quality^3^.

Since the discovery of the essential roles of ipRGCs in the maintenance of circadian rhythm and sleep, there has been a question as to whether there is an association between damage to the RGCs by glaucoma and certain systemic manifestations potentially caused by circadian rhythm disorders. Clinical studies have reported that glaucomatous visual field loss may be correlated with sleep disorders^4^,^5^, and that the specific decline in the pupillary reflex to blue light is correlated with the thickness of the retinal nerve fibre layer (NFL) or visual field loss^6^,^7^,^8^. The NFL is composed primarily of axons of RGCs. Consequently, it has been advocated that ipRGC activity may be associated with decreased sleep quality in patients with glaucoma^9^. However, the underlying cause of sleep disorder in glaucoma patients could also be dependent on psychiatric status, which has been found to be a strong confounding factor in these particular diseases^10^, as well as depression, which has been suggested to be a significant factor in sleep disorder in blindness^11^. Depression is a major cause of sleep disorder, and there is a correlation between depression and the severity of glaucoma^12^,^13^,^14^. In addition, many glaucoma patients have dry eye and problems with the ocular surface caused by topical medications^15^,^16^,^17^. Previously, we found that sleep and mood disorders were most prominent in those with dry eye^10^, a very common disease seriously affecting quality of life^18^.

The aim of the present clinical study was to evaluate the quality of sleep in glaucoma patients to determine correlations between structural changes in the retinal NFL and psychiatric indices for probable sleep and mood disorders. Subjects with dry eye and cataract were excluded from the study because both these conditions are strong confounding factors for sleep and mood disorders^10^.

## Methods

### Study institutions and institutional review board approval

The study was performed in Shinseikai Toyama Hospital (Imizu, Japan), Todoroki Eye Clinic (Tokyo, Japan), and Wakita Eye Clinic (Tokyo, Japan), and was approved by the Institutional Review Board and Ethics Committee of Keio University School of Medicine and Shinseikai Toyama Hospital. Informed consent was obtained from all study participants. The study was performed in accordance with approved guidelines. Study participants, both glaucoma patients and normal controls, were consecutively recruited from patients attending the eye clinics between January and April 2014.

### Participants

The present study was a cross-sectional case-control study. Three hundred and fifty-two participants with suspected glaucoma (based on the results of screening examinations comprising ophthalmoscopy (cupping/disc (c/d) ratio > 0.6) or intra-ocular pressure (IOP; >21 mmHg)) were initially enrolled in the study from patients attending the three eye clinics, located in different parts of Japan. All subjects were examined by board-certified ophthalmologists after undergoing a visual field test (Humphrey Visual Field Analyzer 30–2 standard program; Carl Zeiss, Jena, Germany) and optical coherence tomography (OCT; RC3000; Nidek, Gamagori, Japan).

Two hundred patients were diagnosed as having glaucoma requiring topical medication to reduce IOP. One hundred and thirty-one patients with significant cataract in either eye and/or dry eye were excluded from the study, leaving 69 subjects for analysis. These subjects were further divided into two groups based on mean deviation (MD) values: (1) those with advanced glaucoma (MD less than or equal to −12 dB in the worse eye); and (2) those with moderate glaucoma (MD greater than −12 dB in the worse eye). Seventy-one healthy subjects without visual impairment (<20/25 in either eye), visual field loss (MD greater than −4.0 dB in both eyes, ocular hypertension with elevated IOP (>21 mmHg) in the worse eye), cataract, or dry eye served as the control group.

Diagnostic criteria for glaucoma in the present study included glaucomatous visual field loss less than −4.0 dB MD in the worse eye, an ophthalmoscopic NFL defect, a c/d ratio > 0.6, or elevated IOP (>21 mmHg) requiring topical medication for more than 12 months. Exclusion criteria included coexisting cataract with significant lens opacity disturbing the optical axis that accounted for subjective visual disturbance or decreased visual function, glaucoma surgery, retinal pathology, retinal surgery or photocoagulation affecting the visual field, and dry eye with subjective or objective ocular surface symptoms requiring topical medication.

### Ophthalmological examinations and medications

Ophthalmological examinations included visual acuity, IOP, biomicroscopy, ophthalmoscopy, Humphrey Field Analyzer, and OCT. High myopia was defined as a spherical equivalent less than −5.75 dioptre. OCT was used to determine the thickness of the NFL using in-built software, whereby NFL thickness can be measured on the image as the thickness of the inner three retinal layers, including the inner limiting membrane–inner plexiform layer/inner nuclear layer (ILM-IPL/INL) distance. This value is considered to represent the density of the RGCs. The measured area is given according to hemisphere in both eyes, namely the right or left superior or inferior hemisphere. Mean NFL and NFL in the worst hemisphere (μm) in both eyes were used as representative data for analysis for each patient.

The number and instillation frequency of glaucoma eyedrops were used to estimate medication status. A fixed combination was counted as two medications and instillation frequency was calculated as the total number of times eyedrops were instilled per day. To quantify and evaluate ocular surface toxicity objectively, we used the CVS40/80 (cell viability score) system to express ocular surface cell cytotoxicity in commercial glaucoma eyedrops^19^. This value corresponds precisely to cytotoxicity assay results in cell culture. A CVS40/80 of 100 means no cell death, and, for example, Xalatan^®^ (Pfizer, Tokyo, Japan) and Duotrav^®^ (Japan Alcon Laboratory, Tokyo, Japan) eyedrops have CVS40/80 values of −42 and 83, respectively. In the present study, the ocular surface toxicity of eyedrops was calculated as the sum of [(100–CVS) × (instillation time)] for each eyedrop. The concentration of benzalkonium chloride in eyedrops is not a direct indicator of cytotoxicity because each pharmaceutical component and interactions among many ingredients can alter the cytotoxicity of commercial eyedrops.

### Questionnaires

Participants were asked to complete two validated questionnaires, the Pittsburgh Sleep Quality Index (PSQI)^20^ and the Hospital Anxiety and Depression Scale (HADS)^21^. Each questionnaire was self-administered. The score for each scale was calculated according to separate algorithms and the scores were then analysed. Normal ranges for possible sleep and mood disorders were less than 6 for the PSQI and less than 10 for the HADS. These questionnaires are well validated for screening of sleep and mood disorders. They have been widely used for hospital-based surveys and are easy to answer within 5–10 min, even by those attending eye clinics, because they do not contain difficult questions concerning severe psychiatric disease (e.g. suicide and hallucination).

### Statistical analysis

Data are given as the mean ± SD. Data were analysed using ANOVA, Chi-squared test and Mann–Whitney *U*-test with Bonferroni correction as appropriate. Correlations were evaluated using Pearson product–moment correlation. All analyses were performed using StatFlex^®^ (Atech, Osaka, Japan) and SPSS^®^ version 21 (SPSS Inc., Chicago, IL, USA), with *P* < 0.05 considered significant.

## Results

**Figure 1:**
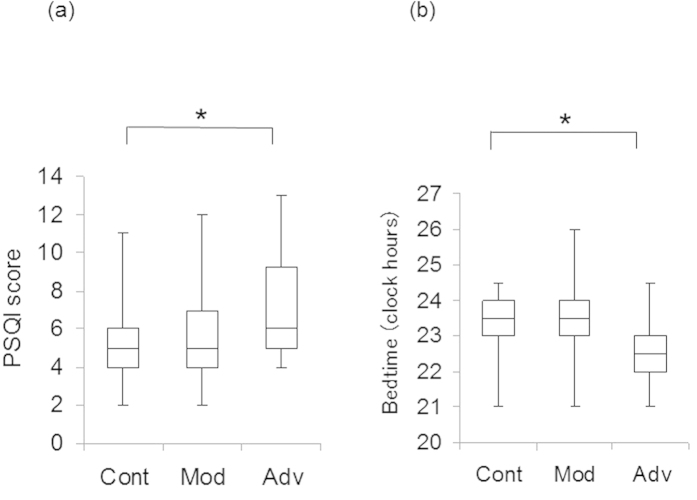
Box plots of Pittsburgh Sleep Quality Index (PSQI) score (**a**) and bedtime (**b**) in normal controls (Cont) and moderate (Mod) advanced (Adv) glaucoma groups. The horizontal lines in each plot indicate median values, the boxes show the interquartile range, and the whiskers indicate maximum and minimum values. PSQI scores and bedtime were significantly worse in the advanced glaucoma group than in the normal controls (*P < 0.05, Mann–Whitney *U*-test with Bonferroni correction).

Patient demographics and univariate comparisons of clinical parameters are given in Tables 1 and 2. Ophthalmological examinations revealed significant differences between advanced glaucoma patients and normal controls in age and ocular parameters. Regarding psychiatric indices and sleep subscales, the PSQI global score was significantly worse in advanced glaucoma patients than normal controls (*P* < 0.05). Forty-one per cent, 58%, and 49% of normal controls and advanced and moderate glaucoma patients, respectively, had probable sleep disorders (PSQI score >5), and 38%, 57%, and 42% of normal controls and advanced and moderate glaucoma patients, respectively, had probable mood disorders (HADS score >9). Box plots of the distribution of PSQI and bedtime are shown in Fig. 1.

**Figure 2:**
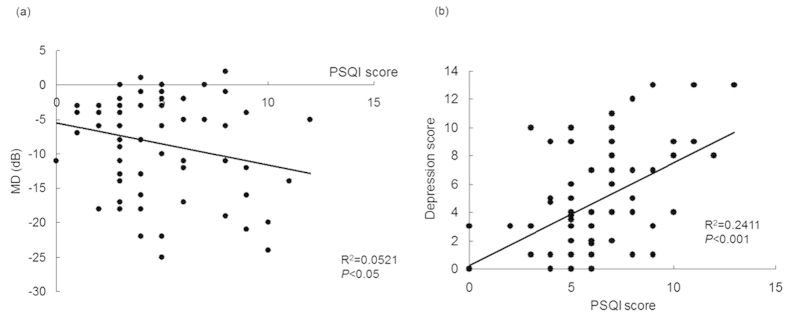
Scatter plot of Pittsburgh Sleep Quality Index (PSQI) scores plotted against (**a**) mean deviation (MD) values, determined using the Humphrey Visual Field Analyzer 30-2 program, and (**b**) the depression subscore on the Hospital Anxiety and Depression scale (HADS) in patients with advanced and moderate glaucoma. Sleep disorders (PSQI global score) were significantly correlated with visual field loss (MD; *R*^2^ = 0.0521, *P* < 0.05, Pearson product–moment correlation) and depression (Depression score; *R*^2^ = 0.2411, P < 0.001).

We next examined which of the variables were independent determinants of mood and sleep. To this end, we used a series of step-wise multivariate linear regression analyses, the variables used given in Table 3. This analysis showed that the global PSQI score was significantly correlated with MD in the worse eye (*P* < 0.05, Pearson product–moment correlation; Table 3; Fig. 2), number of medications (*P* < 0.05), frequency of medication (*P* < 0.05), and HADS-D subscore (*P* < 0.001), but not with NFL thickness or the c/d ratio. Depression was found to be closely associated with the parameters of glaucoma subjects; specifically, the HADS-D subscore was significantly correlated with the MD in the worse eye (*P* < 0.01; Fig. 2), the MD in the better eye (*P* < 0.05), NFL thickness in the worst hemisphere (*P* < 0.05), and IOP (*P* < 0.05).

## Discussion

**Figure 3:**
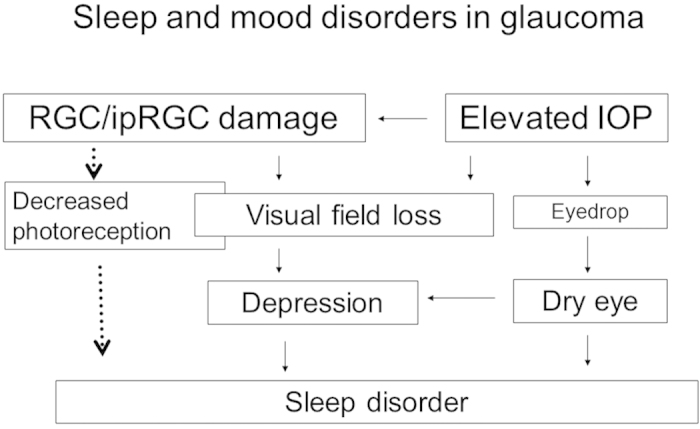
Schematic representation of factors hypothesized to contribute to sleep and mood disorders in glaucoma. Based on the results of the present study, we hypothesize that sleep disorders in glaucoma are associated with visual field loss and medication-related parameters. In addition, many glaucoma patients may develop depression as a result of visual field loss and dry eye symptoms caused by the use of topical glaucoma medications. We did not find any significant relationship between sleep disorders and damage to retinal ganglion cells (RGCs). ipRGC, intrinsically photosensitive RGC; IOP, intra-ocular pressure.

The results of the present study indicate that many patients with advanced glaucoma suffer from sleep disorders. It should be noted that the prevalence of sleep disorders in glaucoma patients is likely to be an underestimate because patients with dry eye and cataract were excluded from the present study, and it is possible that those patients would exhibit a higher prevalence of sleep and mood disorders. Despite the primary aim of the present study being to determine the correlation between sleep quality and ipRGC damage, sleep disorders in glaucoma patients were most strongly correlated with depression score (HADS-D subscale), followed by visual field loss and parameters related to topical medications. It has been reported previously that mood disorders in glaucoma patients are correlated with the severity of visual field loss^12^,^13^,^14^. We speculate that visual field loss may be linked to depression and sleep disorder, and topical glaucoma medications may affect dry eye symptoms and distress, leading to depressive mood^10^ (Fig. 3). The data in the present study did not indicate a significant correlation between sleep disorders and clinical findings of structural damage to the RGCs, even though there was a strong correlation between NFL thickness and visual field loss (data not shown). Although Gracitelli *et al*.^8^,^9^ found a good correlation between ipRGC function and sleep using a specific test to determine ipRGC function, namely the blue light pupil reflex test, a limitation of clinical studies in human ipRGCs is that this cell population is very small and they exhibit very weak electrical responses^22^. The anatomy and physiology of human ipRGCs remain understudied, and it remains unknown to what degree ipRGCs are damaged in glaucoma in humans. The presence of a circadian rhythm disorder has been demonstrated only in an animal model of experimental glaucoma^23^.

Using topical medications itself may be a stressful task for patients with advanced glaucoma and topical medications were correlated with depression subscores in the present study. Most patients used a prostaglandin analogue and were often instructed to use it before bedtime. Patients with advanced glaucoma used a mean of two eyedrops around bedtime, and this task may have disturbed the initiation of sleep. The number and frequency of glaucoma medications are thought to be significantly linked to ocular surface damage because of the presence of both benzalkonium chloride and pharmaceutical ingredients^15^,^16^,^17^. There is a relationship between dry eye and sleep^10^,^24^, and dry eye very frequently develops in glaucoma patients because of the cytotoxic effects of the glaucoma eyedrops^15^,^16^,^17^.

Previous studies have reported an increased prevalence of glaucoma in patients with obstructive sleep apnoea (OSA)^25^,^26^, one of the common diseases underlying sleep disorders. A meta-analysis^25^ and cohort study^26^ found hazard ratios for glaucoma in OSA of 1.65 and 1.67, respectively. An increase in IOP during continuous positive airway pressure therapy for OSA has been suggested to contribute to the development of glaucoma^27^. We did not investigate this issue in the present study, but it should be investigated further because OSA and glaucoma are common diseases in the geriatric population.

Decreased photoreception in the eye has also been suggested to underlie cataract-related sleep disorder^28^,^29^, whereby light transmittance is decreased because of the opacity of the aged crystalline lens. Disturbed vision as a result of cataract leads to decreased physical activity^30^ and so less exposure to blue light during the day. This, in turn, can lead to reduced melatonin secretion at night, as reported in geriatric studies^31^. Recent studies have reported improvements in sleep quality after cataract surgery^32^,^33^.

The present study may be underpowered as a result of its small sample size and this is a major limitation of the study, although appropriate statistical methods were used and analyses were adjusted for several confounding factors after exclusion of dry eye and cataract. The results of the present study need to be confirmed in large cohort studies and using objective methods for sleep evaluation, including polysomnography and actigraphy. Considering structure–function correlations, MD and the thickness of the retinal NFL may not be proportional in glaucoma eyes with a low MD less than −15 dB because of the low reproducibility of visual field measurements and the lower limit of RGC damage^34^,^35^. The clinics participating in the present study did not have pupilometers and ipRGC function was not confirmed by a blue light pupil reflex test.

In summary, the present study showed that visual field loss was correlated with sleep disorder and depression in glaucoma. Structural damage to the RGCs was not significantly associated with sleep disorders. Together, the results indicate that psychological factors may contribute considerably to sleep disorders in glaucoma patients. The findings of the present study may contribute to better management of glaucoma patients with sleep problems and depression.

## Additional Information

**How to cite this article**: Ayaki, M. *et al*. Depressed visual field and mood are associated with sleep disorder in glaucoma patients. *Sci. Rep*. **6**, 25699; doi: 10.1038/srep25699 (2016).

## Figures and Tables

**Table 1:** Patient demographics and univariate comparisons of clinical parameters.

	Control	Advanced glaucoma	*P*-value^A^	Moderate glaucoma	*P*-value^A^
No. subjects	71	24		45	
Age (years)	57.7 ± 15.7	68.4 ± 13.5	0.001*	61.5 ± 15.8	0.01*
No. males/females^B^	29/42	17/7	0.021*	21/24	n.s.
Model 1: Optical parameters and IOP					
LogMAR in the worse eye	−0.03 ± 0.05	0.08 ± 0.30	0.00*	0.01 ± 0.13	n.s.
LogMAR in the better eye	−0.04 ± 0.04	−0.01 ± 0.04	0.03*	−0.57 ± 0.25	n.s.
High myopia (%)	23.3	26.1	n.s	33.3	n.s.
Pseudophakia (%)	14.1	23.3	n.s.	26.2	n.s.
IOP (mmHg)	14.6 ± 4.4	15.9 ± 6.0	n.s.	15.7 ± 4.9	n.s.
Model 2: Visual field and GC structural damage					
MD in the worse eye (dB)	−0.52 ± 1.72	−18.3 ± 5.6	0.00*	−4.4 ± 3.2	0.00*
MD in the better eye (dB)	0.0 ± 1.18	−8.8 ± 8.1	0.00*	−1.9 ± 2.6	0.00*
NFL thickness in the worst hemisphere (μm)	79.7 ± 12.3	54.2 ± 13.5	0.00*	65.5 ± 12.0	0.00*
Mean NFL thickness (μm)	83.6 ± 11.4	64.8 ± 14.7	0.00*	75.7 ± 10.4	0.00*
c/d ratio in the worse eye	0.65 ± 0.09	0.85 ± 0.15	0.00*	0.72 ± 0.13	0.00*
Model 3: Topical medication					
No. medications	–	1.8 ± 1.0		1.4 ± 0.8	
No. instillation (times/day)	–	2.5 ± 2.2		1.8 ± 1.5	
CVS	–	−97 ± 144		−91 ± 61	
Prostaglandin analogue (%)	–	91.7		86.7	
Beta-blocker (%)	–	66.7		40.0	
CAI (%)	–	25.0		11.1	

Unless indicated otherwise, data are given as the mean ± SD.

IOP, intraocular pressure in the worse eye; GC, ganglion cell; MD, mean deviation of Humphrey Field Analyzer 30-2; NFL, nerve fibre layer; c/d ratio, cupping/disc ratio; CVS, cell viability score; CAI, carbonic anhydrase inhibitor.

^A^*P*-values were obtained by Chi-squared test and the Mann–Whitney *U*-test with Bonferroni correction as appropriate; asterisks indicate significant differences (vs Control, *P* < 0.05).

^B^Male = 1, Female = 0.

**Table 2:** Sleep and mood indices of normal controls and glaucoma subjects.

	Control	Advanced glaucoma	*P*-value^A^	Moderate glaucoma	*P*-value^A^
Sleep parameters					
PSQI global score	4.03 ± 1.88	4.81 ± 2.57	0.02*	4.3 ± 2.4	n.s.
Sleep latency score	0.55 ± 0.69	1.20 ± 0.87	0.01*	0.62 ± 0.81	n.s.
Sleep disturbances score	0.72 ± 0.48	0.76 ± 0.54	n.s.	0.71 ± 0.46	n.s.
Sleep efficacy score	0.13 ± 0.41	0.52 ± 0.93	n.s.	0.20 ± 0.51	n.s.
Sleep medication score	0.27 ± 0.83	0.57 ± 1.20	n.s.	0.18 ± 1.10	n.s.
Sleep duration score	1.11 ± 0.82	1.33 ± 0.86	n.s.	1.20 ± 0.84	n.s.
Subjective sleep score	0.94 ± 0.50	1.19 ± 0.75	n.s.	0.87 ± 0.59	n.s.
Daytime dysfunction score	0.31 ± 0.55	0.38 ± 0.59	n.s.	0.53 ± 0.66	n.s.
Bedtime (clock time)	23:26 ± 68 min	22:31 ± 54 min	0.007*	23:28 ± 83 min	n.s.
Mood parameters					
HADS score	9.07 ± 6.36	9.54 ± 6.20	n.s.	8.6 ± 5.8	n.s.
HADS-A subscore	4.64 ± 3.42	4.72 ± 3.11	n.s.	4.5 ± 3.0	n.s.
HADS-D subscore	4.43 ± 3.50	4.82 ± 3.68	n.s.	4.1 ± 3.3	n.s.

Unless indicated otherwise, data are given as the mean ± SD.

PSQI, Pittsburgh Sleep Quality Index; HADS, Hospital Anxiety and Depression Scale; HADS-A, Anxiety subscale; HADS-D, Depression subscale.

^A^*P*-values were obtained by the Mann–Whitney *U*-test with Bonferroni correction; asterisks indicate significant differences (vs Control, *P* < 0.05).

**Table 3:** Step-wise multivariate linear regression analysis of psychiatric indices and ophthalmic parameters in glaucoma patients.

	PSQI		HADS-D subscore		HADS-A subscore	
	β	*P*-value	β	*P*-value	β	*P*-value
Age	−0.20	0.11	−0.25	0.05	−0.32	0.01*
Sex^A^	0.07	0.58	0.19	0.14	0.07	0.60
Model 1: Optical parameters and IOP						
LogMAR in the worse eye	0.05	0.71	0.18	0.17	−0.13	0.32
LogMAR in the better eye	0.01	0.91	0.16	0.24	−0.13	0.31
Myopic refractive errors^B^	0.22	0.17	−0.17	0.28	−0.13	0.40
Phakia/IOL(s)	0.01	0.95	−0.06	0.67	−0.08	0.53
IOP in the worse eye	0.14	0.31	0.30	0.02*	0.12	0.36
Model 2: Visual field loss and GC structural damage						
MD in the worse eye	−0.27	0.04*	−0.40	0.002*	−0.11	0.40
MD in the better eye	−0.22	0.08	−0.28	0.02*	−0.15	0.25
NFL thickness in the worst hemisphere	−0.25	0.14	−0.38	0.02*	−0.19	0.24
Mean NFL thickness	−0.20	0.25	−0.32	0.053	−0.18	0.29
c/d ratio in the worse eye	0.19	0.18	0.17	0.22	0.09	0.54
c/d ratio in the better eye	0.21	0.13	0.18	0.21	0.09	0.52
Model 3: Topical medications						
No. medications	0.27	0.04*	0.30	0.019*	0.17	0.21
No. instillations	0.27	0.04*	0.17	0.003*	0.12	0.37
CVS	−0.16	0.22	0.03	0.82	−0.10	0.43
Model 4: Mood disorders						
HADS score	0.45	0.001*	–	–	–	–
HADS-A subscore	0.43	0.001*	–	–	–	–
HADS-D subscore	0.45	0.001*	–	–	–	–

Variables with an asterisk next to their *P*-value were left in the final models.

*P < 0.05, Pearson product–moment correlation.

All analyses were adjusted for age and sex, as well as degrees of freedom. Parameters were from the worse eye.

IOP, intraocular pressure; IOLs, intra-ocular lenses; GC, ganglion cell; MD, mean deviation on the Humphrey Visual Field Analyzer^®^ 30-2 program; NFL, nerve fibre layer; c/d ratio, cupping/disc ratio; CVS, cell viability score; PSQI, Pittsburgh Sleep Quality Index; HADS, Hospital Anxiety and Depression Scale; HADS-A, HADS Anxiety subscale; HADS-D, HADS Depression subscale.

^A^Male = 1; female = 0.

^B^Spherical equivalent in the eye with the higher myopic refractive error.
